# Detailing the epidemiological and clinical characteristics of chronic lymphocytic leukaemia in Portugal—Results from a population-based cancer registry cohort study

**DOI:** 10.1371/journal.pone.0258423

**Published:** 2021-10-08

**Authors:** Fábio Cardoso Borges, Adriana Ramos, António Lourenço, Maria Gomes da Silva, Ana Miranda, on behalf of the ROR-Sul network

**Affiliations:** 1 National Cancer Registry, Instituto Português de Oncologia de Lisboa Francisco Gentil, EPE, Lisboa, Portugal; 2 NOVA Medical School, Universidade Nova de Lisboa, Lisboa, Portugal; 3 Haematology Department, Instituto Português de Oncologia de Lisboa Francisco Gentil, EPE, Lisboa, Portugal; Mie University Graduate School of Medicine, JAPAN

## Abstract

**Background:**

Chronic lymphocytic leukaemia (CLL) is the most common leukaemia among adults in western countries. Considering the increasing incidence and prevalence of this condition, it is highly relevant to better characterise these patients in Portugal, where data is still scarce.

**Methods:**

To determine incidence, clinical presentation, survival and second malignancies, a population-based historical cohort study was conducted. Cases of interest were identified through the South Region Cancer Registry database and additional data sources. Patients aged ≥18 years, with a confirmed diagnosis of CLL or small lymphocytic lymphoma between January 1^st^, 2013 and December 31^st^, 2014 were included. Patients were followed‐up until death or cut-off date (December 31^st^, 2019).

**Results:**

A total of 496 patients were included and median follow-up time was 5.46 years. Crude incidence rates were 5.03 and 5.22 per 100,000 inhabitants for 2013 and 2014, respectively, and age-adjusted incidence rates were 3.18:100,000 European population for 2013 and 3.35:100,000 European population for 2014. Median age at diagnosis was 71 years and the male/female ratio was 1.40. The majority of patients had leukemic presentation of the disease (86.09%), was diagnosed in Binet stage A (75.58%) and did not present B symptoms (84.01%), anaemia (haemoglobin ≤10g/dL; 90.63%) nor thrombocytopenia (platelet count ≤100 000/μL; 91.73%). Five-year overall survival (OS) rate was 70.53% (95%CI 66.31–74.34) and age, lactate dehydrogenase, Binet stage and a ≥5 Charlson comorbidity index score were independently associated with OS. Standardised-incidence ratios for any second malignancy and cutaneous squamous cell carcinoma were 1.59 (95%CI 1.19–2.08) and 10.15 (95%CI 6.28–15.51), respectively.

**Conclusion:**

Incidence, clinical presentation and survival of CLL Portuguese patients are similar to those reported for other western countries. The increased risk of second malignancies raises concerns and needs adequate clinical watchfulness.

## Introduction

Chronic lymphocytic leukaemia (CLL) is an indolent lymphoproliferative malignancy of CD5+ mature B cells involving peripheral blood, bone marrow and lymphoid organs. It is the most common leukaemia among adults in western countries, with incidence rates ranging between 4 and 6 per 100,000 people per year; in contrast, it is rare in Asia, particularly in Japan and Korea. Incidence increases with age, to more than 30:100,000 inhabitants per year at an age of >80 and, at diagnosis, at least 70% of patients are older than 65 years [1–4].

The diagnosis requires a minimum of 5000 B lymphocytes/μL in the peripheral blood and its clonality needs to be confirmed by flow cytometry [5–7]. Additionally, small lymphocytic lymphoma (SLL) represents a different clinical presentation of the same disease and follows the same management guidelines [3]. This entity is characterised by the presence of lymphadenopathy and/or organomegaly with less than 5000 B lymphocytes/μL in the peripheral blood with a CLL phenotype [5–7].

CLL course is extremely heterogeneous. At diagnosis, the majority of patients are asymptomatic and the disease is detected in a routine blood count. In advanced stages, patients may present anaemia and thrombocytopenia and, scarcely ever, B symptoms. To better stratify patient’s death risk, numerous clinical and biological prognostic predictors have been identified, including Rai and Binet staging systems [8,9], lactate dehydrogenase and β2-microglobulin serum levels [10], tumour genomic aberrations [del(11q), del(13q), del(17p), 12 trisomy)] [11], the mutational status of the immunoglobulin heavy-chain variable region genes (IGHV) [12,13], protein expression (CD38, ZAP-70) [12,14], and the mutational status of the TP53 tumour-suppressor gene [15]. The CLL international prognostic index (CLL-IPI) includes some of these variables and has been validated as predictive of clinical outcomes in various settings in the era of immunochemmotherapy [16].

As other indolent lymphoid disorders, CLL has favourable survival results, with a 5-year relative survival of 80–86% and a death rate of 1.1 per 100,000 people per year [4,17], despite the fact that these patients have an increased risk of developing second malignancies [18,19].

The increasing incidence and prevalence of CLL has augmented the impact of this condition globally. Therefore, it is exceptionally relevant to better understand epidemiology and patients’ characteristics in order to adequate health, social and public health interventions, particularly in Portugal, where this data is still scarce. The aim of this study was to determine the incidence, clinical presentation at diagnosis, survival and occurrence of second malignancies in CLL recurring to a diagnosis cohort from the south region of Portugal.

## Material and methods

### Study design

A population-based historical cohort study was conducted and is reported in accordance with STROBE statement [20].

### Data sources and ethics

The South Region Cancer Registry (ROR-Sul), established in Portugal in 1988, is a population-based cancer registry covering 4.8 million inhabitants from the regions of Lisboa e Vale do Tejo, Alentejo, Algarve and autonomous region of Madeira. ROR-Sul’s database complies with international recommendations stated by the International Agency for Research on Cancer [21] and collects relevant information from time of cancer diagnosis until death. Cancer cases are signalised through institution-based databases (mainly from pathology departments) and numerous variables are semi-automatically transferred into the database (e.g. date of birth, sex, place of residence, topography, morphology, vital status and date of death). Other relevant information, mainly data related to disease stage, prognostic features and treatments are registered manually by trained and experienced personnel at each health institution. The current study was conducted recurring to ROR-Sul’s database.

This study was approved by the *Instituto Português de Oncologia de Lisboa Francisco Gentil* Ethics Committee on September 6th, 2018 (reference: UIC/1201). Informed consent was not required because this is a historical cohort study, all variables used in this study are already part of the ROR-SUL database and are specifically collected to accomplish registry purposes, as dictated by National law.

### Study population

Patients aged 18 years or older diagnosed with CLL or SLL (morphology codes: M9670/3 and M9823/3) [22] between January 1st, 2013 and December 31st, 2014 were included. Cases without histological or immunophenotypical confirmation were excluded. Cases exhaustiveness was maximized per two independent strategies: (a) cases registered in ROR-Sul database with a diagnosis in the study period of non-otherwise specified lymphomas and leukaemias were centrally reviewed and cases of interest were recoded (b) flow cytometry laboratories from public and private hospitals provided results from immunophenotypic analysis conducted during the study period and additional cases of interest were registered in ROR-Sul’s database. Patients were followed-up between diagnosis and death or cut-off date (December 31^st^, 2019).

Information of interest included: age, sex, date of diagnosis, morphology, comorbidities (according to the Charlson index), leukocyte, lymphocyte and platelet counts, haemoglobin, B symptoms, Eastern Cooperative Oncology Group Performance Status (ECOG PS), lactate dehydrogenase, β2-microglobulin, Rai stage, Binet stage, del(17p), del(11q), del(13q), trisomy 12, TP53 mutations, IGHV mutational status, treatment realisation and type of first line treatment, topography and morphology of second malignancies, vital status and date of death/last known contact. Data for variables of interest was centrally validated by the research team.

### Study outcomes

Incidence was determined recurring to crude and age-adjusted (European population) rates. Demographic and clinical characterization was conducted considering information available at diagnosis. Morphology was coded considering a lymphocyte cut-off of 5000x10^6^/L, according to WHO classification [5–7] Comorbidities were collected considering those included in the Charlson comorbidity index (CCI) [23]. Characterisation of chromosomal aberrations per fluorescence in situ hybridization (FISH) and mutational status of the IGHV and TP53 were conducted considering the evaluations performed before the initiation of first treatment.

Overall survival was defined as the time elapsed between diagnosis and death due to any cause. The second malignancies considered were those occurring at least 6 months after CLL/SLL diagnosis (in order to exclude synchronous neoplasms) [24] and included all malignant tumours except basal cell carcinomas.

### Statistical analysis

Data was analysed in a pseudonymized format. Crude incidence rates were computed for the years of 2013 and 2014 using the direct method and considering the resident population in ROR-Sul coverage area publicly provided by Statistics Portugal (INE) [25]. Additionally, age-standardised incidence rates were computed for the European standard population [26].

Demographic and clinical characteristics and second malignancies were summarised using absolute and relative frequencies for categorical variables and median and interquartile ranges (IQR) for continuous variables. We established a cut-off of 20% for missing data and, when the unknown information was lower than the cut-off, it was presented as a complete-case analysis. Otherwise, it was computed for the study population [27]. CCI was calculated according to the original method and summarised with frequencies, mean and standard deviation. Post-hoc bivariate analysis relating Binet stage with sex and age (<65 and ≥65 years) were performed using chi-square test.

Kaplan-Meier estimates were used to compute OS and patients without the event of interest were censored at cut-off date. 5-year survival rates were calculated considering a 95% confidence interval (CI). The log-rank test was used to compare differences between strata. Post-hoc subgroup analyses for OS according to age (<65 and ≥65 years) were performed.

A multivariable proportional hazard regression was used to evaluate the association between variables of interest and OS. Covariates with more than 30% of unknown data were not considered for modeling. Variables included in the multivariable regression were the significant covariates in univariate analysis (cut-off p<0.20) or clinically relevant based on their known prognostic value. Assumptions of the model were verified.

Standardised incidence ratio (SIR) was calculated as the ratio between observed and expected number of second primary malignancies. The expected number of cancers was computed by multiplying ROR-Sul crude incidence rates for 2013 stratified by sex and age groups by stratum-specific person-years at risk of the CLL cohort and summing across strata. For each cancer type, SIR was not calculated to those with less or equal to 5 observed cases. 95%CI was calculated assuming a Poisson distribution for the observed cases.

All statistical analyses were performed using the software Stata version 13.0 [28].

## Results

**Fig 1:**
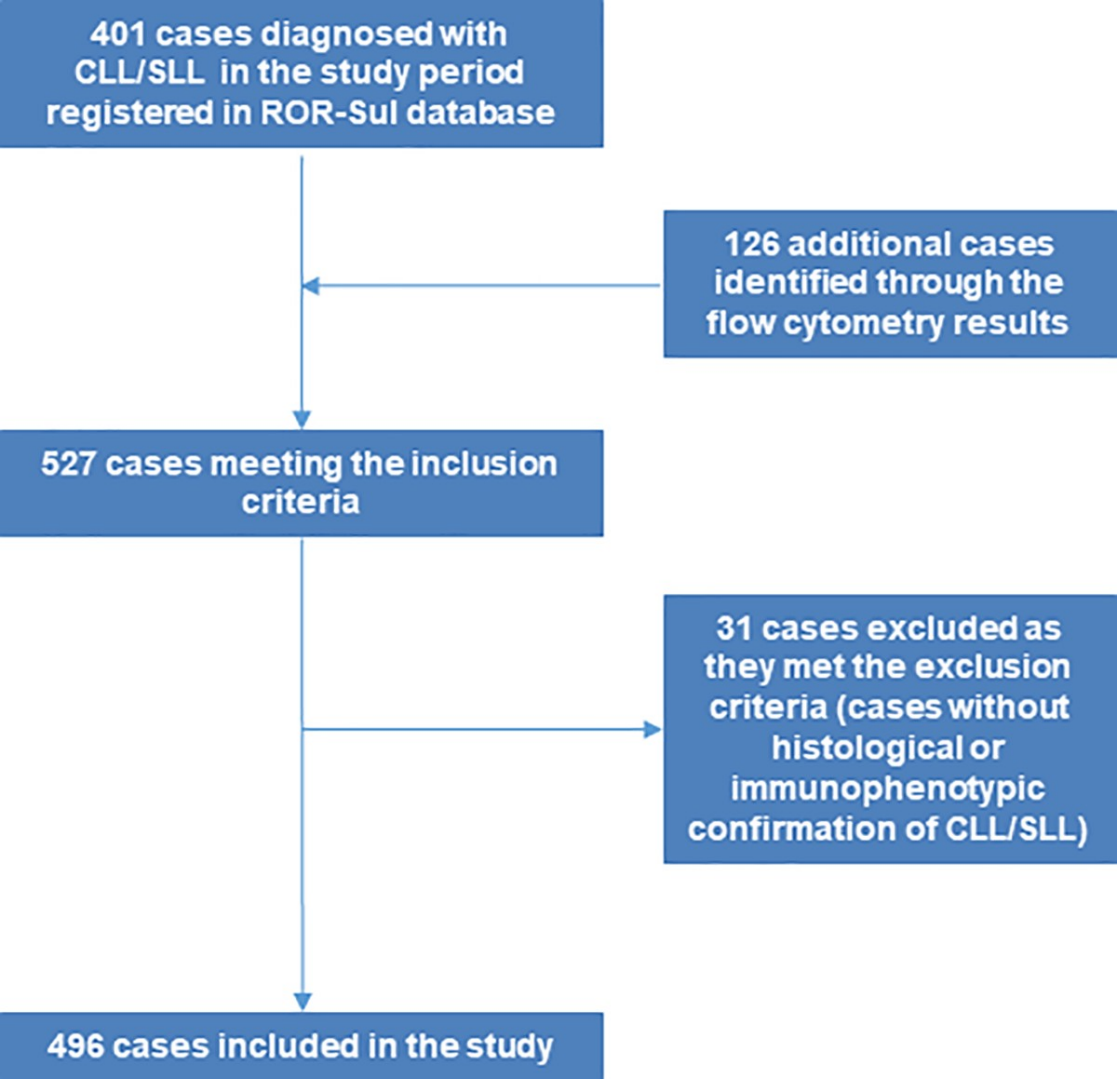
Flowchart for the identification of CLL/SLL cases diagnosed in 2013–2014 in ROR-Sul coverage area.

We first identified in ROR-Sul database 401 cases that met the inclusion criteria. Posteriorly, 126 additional cases were identified through the flow cytometry laboratories’ results and, lastly, 31 cases were excluded as they met the exclusion criteria. The final number of patients included in the study was 496 (Fig 1). Median follow-up of the study population was 5.46 years; only 2 patients (0.40%) were lost to follow-up.

### Incidence and clinical presentation

Crude incidence rates were determined to be 5.03 and 5.22 per 100,000 inhabitants for 2013 and 2014, respectively. Age-adjusted incidence rates (European population), were 3.18:100,000 people for 2013 and 3.35:100,000 people for 2014.

Clinical presentation at diagnosis is shown in Table 1. Median age at diagnosis was 71 years, with 70.77% of patients older than 65 years, and the sex distribution (male/female ratio) was 1.40. The majority of patients had a leukemic picture (80.04%) and were diagnosed in Binet stage A (75.85%). Male sex was associated with more advanced Binet stages (p = 0.044); no statistical significant relationships were found between age and stage (p = 0.290).

**Table 1:** Baseline characteristics of CLL patients diagnosed in 2013–2014 in ROR-Sul coverage area.

Characteristics	n = 496
**Age**	
Median (IQR)	71 (62–78)
≥65 years old, n (%)	351 (70.77)
**Sex, n (%)**	
Female	207 (41.73)
Male	289 (58.27)
Ratio M/F	1.40
**Morphology, n (%)**	
Small lymphocytic lymphoma	99 (19.96)
B-cell chronic lymphocytic leukaemia	397 (80.04)
**Performance status, n (%)**	
0–1	222 (44.76)
≥2	27 (5.44)
Unknown	247 (49.80)
**Comorbidities (CCI)**	
Mean (SD)	0.87 (1.24)
0, n (%)	265 (53.43)
1–2, n (%)	189 (38.10)
3–4, n (%)	32 (6.45)
≥5, n (%)	10 (2.02)
*Unknown*, *n (%)*	0 (0.00)
**Leukocyte count (/μL)**	
Median |(IQR)	16550 (11600–24100)
*Unknown*, *n (%)*	31 (6.25)
**Lymphocyte count (/μL)**	
Median (IQR)	10962.5 (6350–18390)
*Unknown*, *n (%)*	34 (6.85)
**Haemoglobin (g/dL)**	
Median (IQR)	13 (12–15)
≤10 g/dL, n (%)	43 of 459 (9.37)
*Unknown*, *n (%)*	37 (7.46)
**Platelet count (/μL)**	
Median (IQR)	191000 (149000–234000)
≤100 000/μL, n (%)	29 of 455 (6.37)
*Unknown*, *n (%)*	41 (8.27)
**Lactate dehydrogenase, n (%)**	
Normal	242 (48.79)
Elevated	121 (24.40)
Unknown/not evaluated	133 (26.81)
β2**-microglobulin, n (%)**	
Normal	100 (20.16)
Elevated	118 (23.79)
Unknown/not evaluated	278 (56.05)
**B symptoms, n (%)**	
Present	67 (15.99)
Absent	352 (84.01)
*Unknown*	*77 (15*.*52)*
**Rai stage, n (%)**	
0	199 of 472 (42.16)
I/II	230 of 472 (48.73)
III/IV	43 of 472 (9.11)
*Unknown*	*24 (4*.*84)*
**Binet stage, n (%)**	
A	358 of 472 (75.85)
B	73 of 472 (15.47)
C	41 of 472 (8.69)
*Unknown*	*24 (4*.*84)*

**CCI,** Charlson comorbidities index; **IQR,** interquartile range**; M,** male**; F,** female.

At least one chromosomal aberration was evaluated by FISH in 203 patients (40.93%) and the four abnormalities of interest were studied in 198 patients (39.92%). Of these, 62.12% exhibited abnormalities and the most prevalent alteration was 13q deletion (Table 2). In 104 patients there was one aberration, 12 patients had two and 3 patients had three aberrations.

**Table 2:** Prevalence of chromosomal aberrations on CLL patients detected by FISH.

Aberration	n = 496
Not evaluated	293 (59.07%)
Evaluated	203 (40.93%)
No abnormalities	75 of 198 (37.88%)
Chromosomal abnormalities	123 of 198 (62.12%)
Del(13q)	68 of 199 (34.17%)
Trisomy 12	42 of 200 (21.00%)
Del(11q)	22 of 198 (11.11%)
Del(17p)	16 of 199 (8.04%)

The mutational status of the IGHV and TP53 were evaluated in 82 (16.53%) and 39 (7.86%) patients, respectively. With respect to IGHV, 43 patients (52.44%) were hypermutated and 39 (47.56%) were non-hypermutated. TP53 mutation was present in 5.13% of evaluated patients.

### Treatment and survival

During the study period, the majority of patients did not receive any treatment (mostly due to a watch and wait strategy) and 188 patients (37.90%) received at least one therapeutic line for CLL. The most common first-line treatment options were chemmoimmunotherapy (46.28%), including fludarabine, cyclophosphamide, rituximab (n = 49; 26.06%) and bendamustine, rituximab (n = 14; 7.45%), alkylating agent +/- corticosteroids (28.19%) and anthracycline or vinca alkaloids-containing regimens (17.02%).

**Fig 2:**
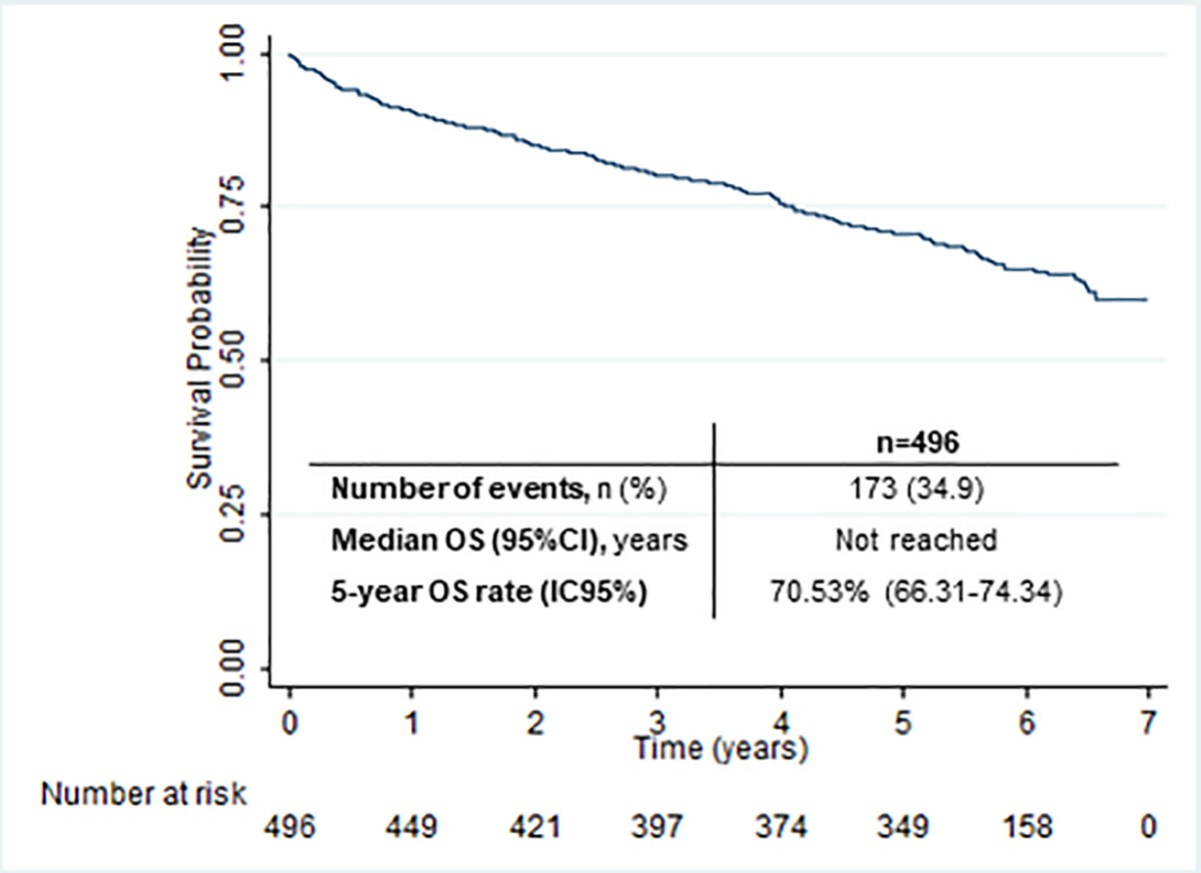
Kaplan-Meier estimate for OS.

OS estimate is presented in Fig 2. Median OS was not reached and 5-year survival rate was 70.53% (IC95% 66.31–74.34).

OS estimate was stratified according to Binet and Rai staging systems (S1 and S2 Figs). Median OS was not reached for the majority of strata although it was reached for high-risk/advanced stages [Binet C: 1.62 years (0.63-not reached); Rai III/IV: 1.62 years (0.64-not reached)]. Additionally, OS was stratified according to age (<65 and ≥65) and, although medians were not reached, the 5-year OS rates were 86.66% (95%CI 83.43–93.63) and 62.62% (95%CI 57.33–67.45) for younger and older patients, respectively.

Univariate and multivariable analysis evaluating the association between baseline characteristics and OS is presented in Table 3. Due to the high number of cases with missing information, β2-microglobulin levels were not included in this analysis. Age, lactate dehydrogenase, Binet stage and a CCI ≥5 score were identified as independent factors associated with OS (p<0.001, p = 0.004, p = 0.002 and p = 0.021, respectively).

**Table 3:** Association between baseline characteristics and OS in CLL patients.

Baseline characteristic	Events	Univariate analysis Crude HR (95%CI)	*p-value*	Multivariable analysis Adjusted HR (95%CI) n = 343, 122 events	*p-value*
**Age** (continuous variable)	173/496	1.10 (1.08–1.11)	<0.001	1.10 (1.07–1.12)	<0.001
**Sex** Male vs female (ref)	173/496	0.93 (0.69–1.25)	0.617	1.35 (0.91–1.99)	0.136
**Leukocyte count** (continuous variable)	166/465	≈1 (1–1)	0.058	1.01 (0.99–1.03)	0.167
**Lymphocyte count** (continuous variable)	165/462	≈1 (1–1)	0.077	0.99 (0.97–1.01)	0.227
**Morphology** SLL vs CLL (ref)	173/496	1.38 (0.97–1.96)	0.073	1.34 (0.86–2.09)	0.194
**Lactate dehydrogenase** Elevated vs normal (ref)	130/363	2.02 (1.43–2.86)	<0.001	1.74 (1.19–2.55)	0.004
**B symptoms** Present vs absent (ref)	141/419	0.53 (0.13–0.93)	0.009^1^	-	-
**Binet stage**	160/472				
B vs A (ref)		1.59 (1.07–2.38)	0.023	2.08 (1.30–3.33)	0.002
C vs A (ref)		3.66 (2.37–5.63)	<0.001	2.39 (1.36–4.21)	0.002
**CCI**	173/496				
1–2 vs 0 (ref)		1.40 (1.02–1.93)	0.04	1.36 (0.92–2,01)	0.128
3–4 vs 0 (ref)		2.19 (1.28–3.75)	0.004	0.94 (0.49–1.82)	0.858
≥5 vs 0 (ref)		3.92 (1.81–8.51)	0.001	2.99 (1.18–7.56)	0.021

**CCI,** Charlson comorbidities index; **CI,** confidence interval**; CLL,** Chronic lymphocytic leukaemia; **SLL,** Small lymphocytic lymphoma.

^1^This variable was not included as a covariate because it violated the assumption of proportional hazards.

### Second malignancies

A total of 53 second malignant tumours were identified in 46 patients, which corresponds to a cumulative incidence of 10.69%. The most common second malignancies were cutaneous squamous cell carcinoma (CSCC) (n = 21), prostate cancer (n = 5) and malignant tumours of the central nervous system (n = 4) (S1 Table). The risk of any second primary cancer in CLL patients was increased comparing to the general population [SIR = 1.59 (95%CI 1.19–2.08)]. Site-specific SIR was calculated to CSCC and estimated to be 10.15 (95%CI 6.28–15.51).

## Discussion

Cancer registries are important tools in the generation of epidemiological data. To the best of our knowledge, this is the first population-based study that aimed to characterise the incidence, clinical presentation, survival and the occurrence of second malignancies in CLL patients in Portugal. As this condition is particularly incident and prevalent in western countries, with a significant impact on both social and health systems, it is of utmost relevance to better understand its epidemiology and patients’ characteristics.

With respect to cases completeness, it is worth noticing that, in this study, patients identified through flow cytometry laboratories represent over 25% of the total cases; in fact, a 20–30% under-reporting of CLL cases for other population-based cancer registries was already described [29–31]. Thus, the use of additional information sources as a routine strategy is mandatory in order to provide accurate incidence and survival data for CLL/SLL. Nevertheless, it should be highlighted that the present work contributed to an accurate CLL/SLL incidence and survival determination in Portugal and illustrates the importance of adapting CLL/SLL epidemiologic surveillance to provide reliable and comparable data.

Incidence rates were comparable to other western countries, including the United States of America (USA), Canada and European countries such as Spain and Sweden, but contrasted with the incidence in the United Kingdom and Belgium (estimated at 6:100,000 European population) [29,32–37], which may be related to differences in characteristics of the population and/or risk factors. Differences were also observed when comparing with data from Eastern Europe, Russia and Asian countries, where it a lower incidence of this malignancy has been described. Age at diagnosis and sex ratio were also comparable to other western countries [32,35,37].

The general outcomes of this population, predominantly of early stage CLL and in patients with good performance status, were very favourable. Median OS was not reached, as could be expected due to the relatively short follow-up duration in an indolent malignancy (5.46 years). Nevertheless, the 5-year OS rate is concordant with survival results reported for other developed countries (mainly western countries such as Germany, Sweden, Norway) and contrasts with those from developing countries [17,34,37–39].

The analysis of the clinical characteristics at diagnosis showed similarities to data reported for other developed countries [40–42] and diverges from data from developing countries, in which patients are diagnosed in more advanced/high risk stages, with higher prevalence of anaemia and thrombocytopenia [38,42,43]. The proportion of patients with unknown information for β2-microglobulin (56.05%) should be noted; although it is independently associated with OS and response duration [44], it was not systematically evaluated in the study population. It is also imperative to mention that the majority of patients (53.43%) did not present any of the comorbidities of the CCI and had ECOG PS≤2. Even though other authors reported the absence of comorbidities at diagnosis in 70% of CLL patients [45], we recognise that the application of CCI in CLL is limited. The *Cumulative Illness Rating Scale* (CIRS), not available in ROR-Sul database, is more frequently used [46]. Another issue that might have contributed to these results is the retrospective collection of this information, which could have led to information bias.

Chromosomal aberrations were evaluated by FISH in 40.93% of patients before treatment, which represents a limitation for data interpretation. Nonetheless, this proportion of evaluated patients was expected as, during the study period, the recommendations to conduct this characterisation were still limited and had no impact in therapeutic decisions in our country [47]. Generally, the prevalence of these alterations is in agreement with studies performed in other populations, although it should be noted the higher proportion of patients without FISH abnormalities (37.69% vs 15–25%) [17,18,39,43,45]. As mentioned before, this analysis and interpretation of the results have limitations and, to achieve robust conclusions, more studies should be performed, ideally prospective, with larger series of patients.

Treatment characterisation showed that a watch and wait strategy was chosen for the majority of the cases, as recommended in current guidelines and as it would be expected considering the follow-up duration (5.46 years). First-line treatment options generally agreed with international recommendations for that time period, although regimens containing anthracyclines and vinca alkaloids were also unexpectedly used and should be explored in additional/future research.

OS stratification per Rai and Binet staging systems, as well as the multivariable analysis, apparently validate its use in this population (p<0.001 and p = 0.004, respectively). Median OS was reached for Binet stage C [1.62 years (95%CI 0.63 –not reached)] and Rai stage III/IV [1.62 years (95%CI 0.64 –not reached)] strata. Although these results differ from others reported in the literature (e.g. Binet C having a median OS of 7.9 years [17]), the interpretation of this data should be cautious due the low number of patients in these stratum (n = 41) and the short follow-up duration, which contributed to a less precise estimate, as can be appreciated looking at the CI amplitude. Additionally, treatment options, that may have had an important impact on survival outcomes, were only restrictedly analysed in this series.

Our study confirmed age, lactate dehydrogenase and Binet stage as being independently associated with OS. In our analysis, a CCI score ≥5 was independently associated with OS although CCI 1–4 strata were not. This result is in agreement with those reported in the literature per other authors [48–50].

Regarding second malignancies, the proportional incidence of CSCC, malignant tumours of the central nervous system, myelodysplastic syndromes and malignant melanomas of the skin is superior to that reported to the general population. The SIR observed to any second cancer identified a 1.59 fold increased risk and this result is concordant with data from other series [51–53]. Site-specific SIR calculated for CSCC revealed a 10.15 increased risk for the development of this tumour. There are few studies in the literature that evaluated the risk of developing CSCC after CLL. Zheng et al. observed a 7.63 fold increased risk (95%CI 7.06–8.25) for the Swedish population and Ishdorj and colleagues found a five-fold increased risk in the Canadian population [54,55], inferior to our results although with coincident CI. It should be noted that our observations arise from population-based data, which contributed to cases exhaustiveness of both CLL and second malignancies and consequently to the accuracy of the estimates. However, we admit that the number of patients included in this study may have contributed to a less precise estimate as shown by the CI amplitude. Nevertheless, these results raise concerns and highlight the importance of adequate clinical watchfulness during the follow-up of CLL patients.

This study has various strengths: it was conducted at the population level, included patients from both private and public hospital institutions and cases of interest were signalised through several data sources, which constitute major contributions to the external validity of our results. It is worth noting the central procedures for data accuracy and validation, which contributed to the internal validity of our work. It is also important to highlight the proportion of follow-up completeness (99.6%), extremely relevant to characterise the outcomes of interest. Nonetheless, there are also some limitations, namely the proportion of unknown/not evaluated information for some of the variables of interest and the absence of some other relevant data (e.g. autoimmune phenomena), which is inherent to observational/registry studies, but can lead to information bias. Additionally, it is worth mentioning the possible misclassification bias of some of the variables of interest used to analyse the outcomes. However, procedures were implemented to its minimisation, namely the central validation of the information for those variables. Another limitation concerns the relatively short follow-up considering the characteristically indolent evolution of this disease; hence, a longer follow-up would be important to achieve more robust results.

## Conclusions

Our study confirms that epidemiological and clinical characteristics of Portuguese CLL patients are concordant to those reported from other western countries. Clinical outcomes, measured by 5-year overall survival rates, are also similar to other western countries. The increased risk of second malignancies raises concerns and needs adequate clinical watchfulness.

## Supporting information

S1 Fig Kaplan-Meier estimate for OS stratified per Rai stage. (TIF)

S2 Fig Kaplan-Meier estimate stratified per Binet stage. (TIF)

S1 Table Second malignancies occurred in CLL patients. **CLL,** Chronic lymphocytic leukaemia; **CNS,** Central nervous system; **GIST,** Gastrointestinal stromal tumour.
(DOCX)
