# Supplementary figures and images for: Detailing the epidemiological and clinical characteristics of chronic lymphocytic leukaemia in Portugal—Results from a population-based cancer registry cohort study
Source: PLoS One. 2021 Oct 8;16(10):e0258423. doi: 10.1371/journal.pone.0258423 (PMC8500441; doi:10.1371/journal.pone.0258423)

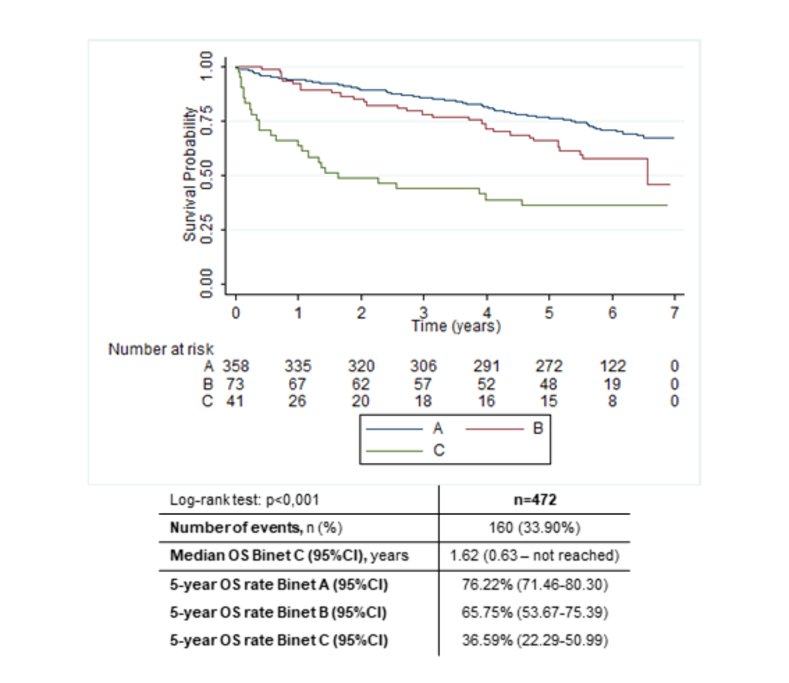

Supplement: S1 Fig — (TIF) [file pone.0258423.s001.tif]

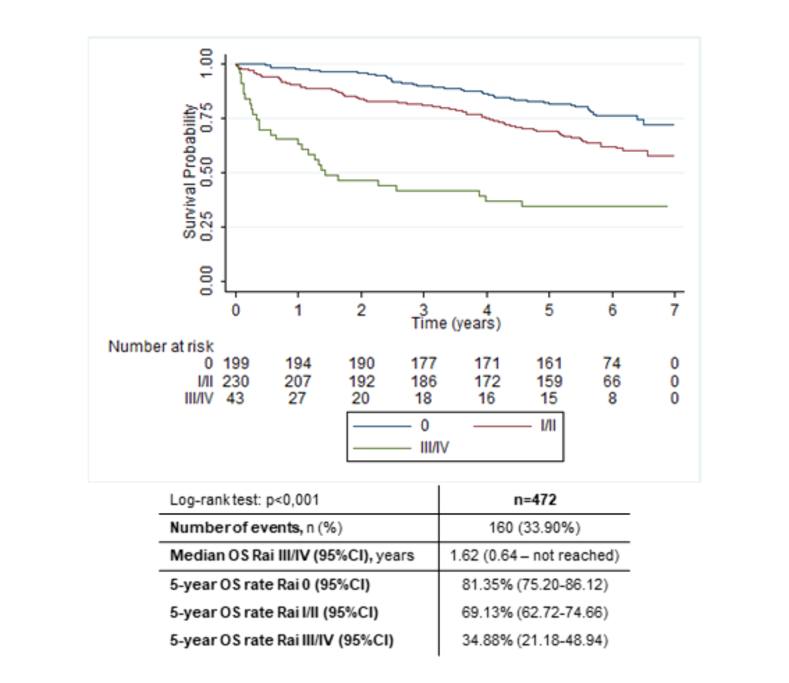

Supplement: S2 Fig — (TIF) [file pone.0258423.s002.tif]
