# Supplementary material for: Detailing the epidemiological and clinical characteristics of chronic lymphocytic leukaemia in Portugal—Results from a population-based cancer registry cohort study
Source: PLoS One. 2021 Oct 8;16(10):e0258423. doi: 10.1371/journal.pone.0258423 (PMC8500441; doi:10.1371/journal.pone.0258423)
Supplement: S1 Table — CLL, Chronic lymphocytic leukaemia; CNS, Central nervous system; GIST, Gastrointestinal stromal tumour. (DOCX) [file pone.0258423.s003.docx]

**S1 Table. Second malignancies occurred in CLL patients.**

| **Malignant tumour** | **n** | **Proportional incidence** | **n=496** |
| --- | --- | --- | --- |
| Cutaneous squamous cell carcinoma | 21 | 39.62% | 4.23% |
| Prostate cancer | 5 | 9.43% | 1.01% |
| Malignant tumours of the CNS | 4 | 7.55% | 0.81% |
| Breast cancer | 3 | 5.66% | 0.60% |
| Myelodysplastic syndrome | 3 | 5.66% | 0.60% |
| Stomach cancer (carcinoma, adenocarcinoma e GIST) | 3 | 5.66% | 0.60% |
| Lung cancer | 2 | 3.77% | 0.40% |
| Malignant melanoma of the skin | 2 | 3.77% | 0.40% |
| Thyroid cancer | 2 | 3.77% | 0.40% |
| Uterus cancer (adenocarcinoma e cystadenocarcinoma) | 2 | 3.77% | 0.40% |
| Acute myeloid leukaemia | 1 | 1.89% | 0.20% |
| Bladder cancer | 1 | 1.89% | 0.20% |
| Colon cancer | 1 | 1.89% | 0.20% |
| Liver cancer | 1 | 1.89% | 0.20% |
| Orbital cancer | 1 | 1.89% | 0.20% |
| Rectum cancer | 1 | 1.89% | 0.20% |
| Total | 53 | 100.00% | - |

**CLL,** Chronic lymphocytic leukaemia; **CNS,** Central nervous system; **GIST,** Gastrointestinal stromal tumour;
